# Supplementary material for: MetaQTL: a package of new computational methods for the meta-analysis of QTL mapping experiments
Source: BMC Bioinformatics. 2007 Feb 8;8:49. doi: 10.1186/1471-2105-8-49 (PMC1808479; doi:10.1186/1471-2105-8-49)
Supplement: Additional File 7 — MetaQTL Package : jar file and tutorial. This Zip archive contains both the MetaQTL JAR file and the files of the tutorial. [file 1471-2105-8-49-S7.zip › org.inra.metaqtl/doc/org/thalia/bio/util/BioCrossTypes.html]

BioCrossTypes


|  |  |  |
| --- | --- | --- |
| **Overview** \| **Package** \| **Class** \| **Use** \| **Tree** \| **Deprecated** \| **Index** \| **Help** | |  |
| PREV CLASS   **NEXT CLASS** | **FRAMES**    **NO FRAMES**     **All Classes** |  |
| SUMMARY: NESTED \| FIELD \| CONSTR \| METHOD | DETAIL: FIELD \| CONSTR \| METHOD |  |


---


## org.thalia.bio.util Class BioCrossTypes

```
java.lang.Object
  org.thalia.bio.util.BioCrossTypes
```

---

``` public final class BioCrossTypes extends java.lang.Object ```

**Author:**
:   jveyrier
    TODO To change the template for this generated type comment go to
    Window - Preferences - Java - Code Style - Code Templates

---

| **Field Summary** | |
| --- | --- |
| `static int` | `gen` |
| `static int` | `type` |


**Constructor Summary**

`BioCrossTypes()`


| **Method Summary** | |
| --- | --- |
| `static java.lang.String` | `crossToString(int type1, int gen1)` |
| `static void` | `parseCross(java.lang.String str)`             Parse the given string and return if valid the type of cross and the number of generations. |

**Methods inherited from class java.lang.Object**

`clone, equals, finalize, getClass, hashCode, notify, notifyAll, toString, wait, wait, wait`

**Field Detail**

### type

```
public static int type
```

---


### gen

```
public static int gen
```


**Constructor Detail**

### BioCrossTypes

```
public BioCrossTypes()
```


**Method Detail**

### parseCross

```
public static void parseCross(java.lang.String str)
```

:   Parse the given string and return if valid the type of
    cross and the number of generations.
    Design Code Example
    Backcross j times to Pi BCi BC1
    Selfed Generation i intercross SFi SF3
    Randomly mated generation i intercross RFi RF2
    Double Haploid RI0 RI0
    RIL via selfing RI1 RI1
    RIL via sib-mating RI2 RI2
    IRI via selfing + i randomly mated gen IRI0i IRI04
    IRI via sib-mating + i randomly mated gen IRI1i IRI13

    :   **Parameters:**: `str` -

---


### crossToString

```
public static java.lang.String crossToString(int type1,
                                             int gen1)
```

:   **Parameters:**: `type2` -: `gen2` - **Returns:**


---


|  |  |  |
| --- | --- | --- |
| **Overview** \| **Package** \| **Class** \| **Use** \| **Tree** \| **Deprecated** \| **Index** \| **Help** | |  |
| PREV CLASS   **NEXT CLASS** | **FRAMES**    **NO FRAMES**     **All Classes** |  |
| SUMMARY: NESTED \| FIELD \| CONSTR \| METHOD | DETAIL: FIELD \| CONSTR \| METHOD |  |


---
